# Icariin synergizes therapeutic effect of dexamethasone on adriamycin-induced nephrotic syndrome

**DOI:** 10.1186/s40001-022-00973-9

**Published:** 2023-01-27

**Authors:** Juan Lv, Guozhong Xue, Yunxia Zhang, Xinbin Wang, Enlai Dai

**Affiliations:** 1 Department of Integrated Traditional Chinese and Western Medicine, Gansu University of Chinese Medicine, Lanzhou, 730000 Gansu China; 2 Department of Nephrology, Affiliated Hospital of Gansu University of Chinese Medicine, Lanzhou, Gansu China; 3 Department of Neurology, Gansu Provincial Hospital of TCM, Lanzhou, Gansu China

**Keywords:** Adriamycin, Autophagy, Podocyte injury, Nephrotic syndrome

## Abstract

**Background:**

Glomerular damage is a common clinical indicator of nephrotic syndrome. High-dose hormone treatment often leads to hormone resistance in patients. How to avoid resistance and improve the efficiency of hormone therapy draws much attention to clinicians.

**Methods:**

Adriamycin (ADR) was used to induce nephropathy model in SD rats. The rats were treated with dexamethasone (DEX), icariin (ICA), and DEX + ICA combination therapy. The changes in urinary protein (UP), urea nitrogen (BUN), and serum creatinine (SCR) contents in rats were detected by enzyme-linked immunosorbent assay (ELISA), and the degree of pathological injury and the expression level of podocin were detected by HE staining and immunohistochemistry, to test the success of the model and the therapeutic effects of three different ways. The effect of treatments on podocytes autophagy was evaluated via transfection of mRFP-GFP-LC3 tandem adenovirus in vitro*.*

**Results:**

The contents of UP, SCR, and BUN were significantly increased, the glomerulus was seriously damaged, and the expression of Nephrosis2 (NPHS2) was significantly decreased in the ADR-induced nephrotic syndrome rat model compared to that of the control group. DEX, ICA, and the DEX + ICA combined treatment significantly alleviated these above changes induced by ADR. The combined treatment of DEX + ICA exhibited better outcome than single treatment. The combined treatment also restored the podocyte autophagy, increased the expression of microtubule-associated protein light-chain 3II (LC3II), and reduced the expression of p62 in vitro. The combined treatment protects podocytes by mediating the PI3K/AKT/mTOR (rapamycin complex) signaling pathway.

**Conclusion:**

ICA enhances the therapeutic effect of DEX on the nephrotic syndrome.

## Introduction

Nephrotic syndrome (NS) is not an independent disease, but a group of clinical symptoms in glomerular disease. It is a common and multiple disease in nephrology. NS is characterized by proteinuria, edema, hyperlipidemia, and hypoalbuminemia [1], which is a kind of chronic kidney disease with a large loss of urinary protein due to the damage of the glomerular basement membrane. Hormones are effective drugs that inhibit immunity, effectively clear immune complexes (IC), and reduce urinary protein [2]. At present, the preferred drug for clinical induction of remission of the disease is an adrenocortical hormone, such as DEX. On the basis of symptomatic treatment and control of complications, medium- and long-term hormone therapy are often used. Although it has achieved a good curative effect, it is prone to recurrence or hormone resistance, a secondary infection caused by large doses of the hormone, Cushing’s syndrome, and other toxic side effects [3], which have seriously affected daily life and life health of patients. Therefore, it is urgent to develop new therapeutic drugs and methods.

Podocytes are end-stage differentiated renal epithelial cells located in the outer layer of the glomerular basement membrane, which play an important role in maintaining the integrity of the glomerular filtration barrier and normal glomerular filtration function [4]. Once the podocytes are damaged, the regeneration ability and repair ability of podocytes are very limited. After podocyte injury, its migration ability increases abnormally, and it will fall off from the glomerular basement membrane, resulting in the damage of the glomerular filtration barrier [5], resulting in proteinuria. Studies have found that drugs that exert pharmacological activity through immunosuppression, such as GCS, can act directly on podocytes [6]. Therefore, the methods of protecting podocytes, such as alleviating podocyte damage and maintaining the integrity of podocyte structure, are very important for the treatment of chronic kidney diseases such as NS.

ICA is one of the main effective components of epimedium flavonoids extracted from traditional Chinese medicine epimedium, with a relative molecular weight of 676.662, which is difficult to dissolve in water. ICA has a variety of biological activities, such as anti-tumor [7], anti-osteoporosis [8], anti-depression [9], improving cognitive function of Alzheimer's disease [10], etc. In addition, studies have shown that ICA has a certain effect on diabetes [11] and diabetes complications [12], but the role of ICA and Dex in the comprehensive treatment of nephrotic syndrome has not been studied, and the molecular mechanism of its improvement of nephrotic syndrome in patients is not clear. Therefore, this experiment mainly investigates the effect of ICA and DEX on the treatment degree of ADR-induced nephrotic syndrome and podocyte autophagy, so as to provide theoretical basis and scientific basis for revealing the pathological mechanism of nephrotic syndrome complications and research and development of methods.

## Materials and methods

### Establishment of ADR-induced nephrotic syndrome rat model

In this study, the 7-week-old male Sprague–Dawley (SD) rat were purchased from Laboratory Animal Center, Lanzhou Veterinary Research Institute, Chinese Academy of Agricultural Sciences. According to the experimental design, the rats were divided into five groups: control group (control), model group (ADR, No. D8740, Solarbio, Beijing, China), DEX group (DEX, No. D8040, Solarbio, Beijing, China), icariin group (ICA, No. SI8010, Solarbio, Beijing, China), and DEX combined with icariin group (combination). Control is a normal and healthy SD rat, and the rest of the groups were established based on the adriamycin (ADR)-induced nephrotic syndrome rat model.

The protocol of the ADR-induced nephrotic syndrome model is based on previous studies [13, 14]. Briefly, 8-week-old male SD rats (200 ± 20 g), were injected with ADR (15 mg/kg) through the tail vein [15]. The urine protein and creatinine were measured weekly. According to previous studies, the model is considered successful when the urine protein level is higher than 30 mg/24 h [16]. Two weeks after modeling, the model rats were randomly divided into four treatment groups (*n* = 8 for each)—model, DEX (5 mg/kg/d), ICA (60 mg/kg/d) [17], and combination. Animals from the control and model groups received injections of the same amount of saline as those in the treatment group. After 6 weeks of treatment, the rats were euthanized by cervical dislocation after venous blood collection. Tissues and serum were collected for subsequent experiments.

### Urinary protein, creatinine and albumin, BUN, and serum creatinine

After the tail vein injection of ADR, the rats were placed in a metabolic cage and collected the 24 h urine every 2 weeks. Six weeks after drug administration, rats were anesthetized by intraperitoneal injection of 10% chloral hydrate solution (0.3–0.4 mL/100 g), and 6–8 mL of blood were collected by cardiac phlebotomy. The levels of urine protein, Scr, and BUN were measured with the automatic biochemical analyzer (cobasc 701, Roche).

### Hematoxylin–eosin (HE) staining

HE staining was conducted according to routine protocols [18]. Briefly, put the slices into toluene I and toluene II for treatment for 10 min successively, put the slices after xylene treatment into absolute ethyl alcohol I and absolute ethyl alcohol II for soaking treatment for 5 min, then soak them in different concentrations of alcohol (95%, 90%, 80%, 70%) for 5 min successively, wash the alcohol with distilled water, and put the cleaned slices into hematoxylin and eosin staining solution to dye the nucleus and cytoplasm, and finally dehydrate and seal the slices; image acquisition was carried out through Soptop, Rx50 microscope (sunny optical technology (Group) Co., Ltd., China, Beijing). The pathological changes of glomerulus injury were observed and the glomerulus injury score analysis was performed as described by Paller et al. [19]^.^

### Immunohistochemical staining

The frozen tissue sections were blocked with the goat serum for 30 min for NPHS2 (No. ab216341, abcam, China) antibody incubation. Following SP reaction, DAB staining, counterstaining, dehydration, and transparency, the slide was mounted and the slides were then examined and photographed using a SOPTOP RX50 fluorescence microscope. The DAB staining was analyzed by Image-Pro Plus 6.0 software.

### Cell culture

Rat renal podocytes were purchased from BeNa Culture Collection (BNCC, Henan, China) and cultured in high glucose Dulbecco’s modified Eagle medium (DMEM) containing 20U/mL γ-IFN, 10% fetal bovine serum (FBS), and 1% penicillin. Renal podocytes differentiate into mature differentiated cells after 2 weeks. The culture medium was replaced with high glucose DMEM, 10% FBS, and 1% penicillin. All cell experiments were performed when podocytes were differentiated and matured.

### Adriamycin-induced podocytes injury model in vitro

The differentiated and mature cells were seeded in 96-well plates. After 24 h of incubation, the plates were added with ADR in different concentrations (0.5 µmol/L, 1.0 µmol/L, 2.0 µmol/L, 5 µmol/L, and 10 µmol/L) for 12 h, 24 h, and 48 h. The cell apoptosis rate under different treatment conditions was measured to determine the optimal time and concentration of ADR for injury model establishment.

### MTT method

MTT evaluated the half maximal effective concentration (EC50) of DEX and ICA to determine the following experiment's optimal concentration. Four drug concentrations (0.1 µmol/L, 1 µmol/L, 10 µmol/L, and 20 µmol/L) were selected to treat the ADR-induced model. After 24 h of treatment, MTT was added to plate. The OD value was measured at 490 nm. The cell growth rate was calculated following different drug concentrations.

### mRFP-GFP-LC3 tandem adenovirus infection and experimental grouping

Based on the above MTT experiment results, an ARD podocyte injury model was established in vitro. According to the previous study [20, 21], mRFP-GFP-LC3 tandem adenovirus was used to infect rat podocyte nuclei to observe the changes of autophagy after DEX and/or ICA treatment. The experiment was divided into four groups: the blank control group (without ADR treatment); ADR injury model group pretreatment for 48 h; combination group and combination + chloroquine (CQ) group treatment for 48 h. The concentration of ADR and the combined treatment concentration of DEX (10 µM) and ICA (10 µM) were determined based on the previous MTT experiments. The concentration of CQ was 10 µmol/L.

### Western blot

Total proteins were extracted from cells in different treatment groups and determined the concentration. SDS-PAGE separated a total of 20 µg protein samples. The protein was transferred to the activated PVDF membrane using the Bio-rad wet transfer system, then blocked with 5% BSA phosphate buffer for 1 h at room temperature. Next, the membrane was incubated with primary antibody overnight at 4 °C followed by the HRP-labeled secondary antibody incubation at room temperature for 1 h. The relative expression of the target protein was visualized in ECL and analyzed with Image J software.

### Statistical analysis

Image-Pro Plus 6.0 was used to analyze the images. The experimental data were analyzed by SPSS 20.0. The figures were produced with GraphPad Prism 7.0. All data were expressed as mean ± SD. One-way ANOVA was used to compare statistical differences among multiple groups, and the independent student’s *t *test was used to compare statistical differences between two groups. *P* < 0.05 was considered statistically significant, *P < 0.05, **P < 0.01. n.s. indicates no significant difference.

## Results

### Icariin synergizes with DEX alleviateadriamycin nephrosis

**Fig. 1:**
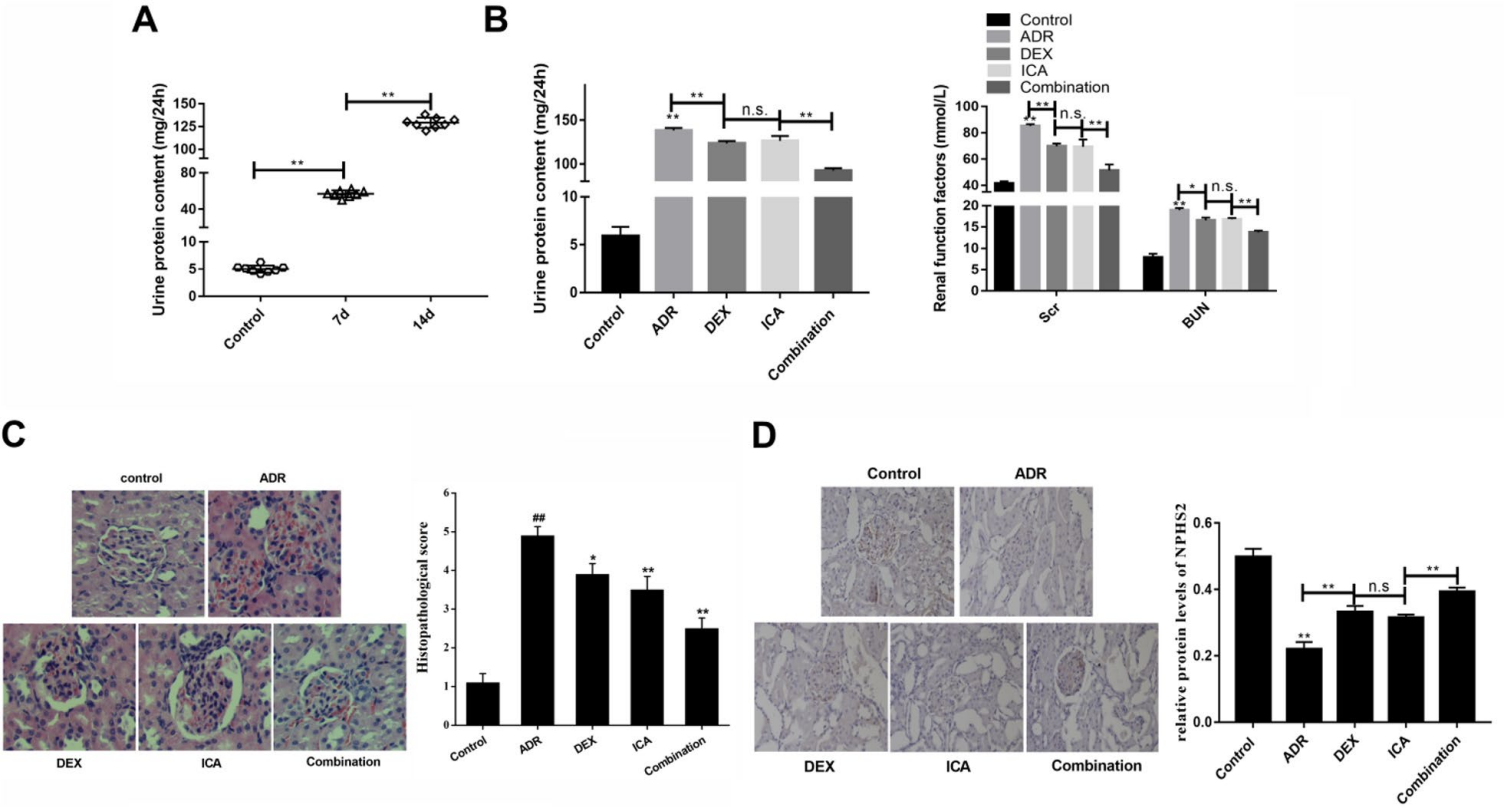
The therapeutic effect of icariin and DEX on nephrotic syndrome model. **A** Changes of urine protein in adriamycin-induced nephrotic syndrome model; **B** UP, Scr, and BUN levels were regulated by icariin and DEX; **C** H&E was used to evaluate the glomerulus injury; **D** immunohistochemical assay for podocyte injury

One of the main features of ADR-induced nephrotic syndrome is the appearance of massive proteinuria. It was found that the rats showed clinical symptoms after 2 weeks of tail vein ADR injection. The physical signs of Yang deficiency in traditional Chinese medicine included weight loss, fatigue, and increased urine protein (Fig. 1A). The DEX, ICA, and combination treatments all showed beneficial effects and significantly relieved proteinuria. Compared with model, the levels of Scr and BUN reduced following combination therapy (Fig. 1B). From the histopathology, we can see that the glomeruli in the model group were obviously enlarged, the glomerulus had adhered, and there were more inflammatory cell infiltrations. The glomeruli and tubules in the control group are clearly visible (Fig. 1C). The NPHS2 expression, a podocyte marker, was upregulated by combination treatment, which alleviated ADR-induced foot process loss (Fig. 1D). The changed NPHS2 expression also indirectly illustrates the success of our model. After the interference of DEX, ICA, and combination, the glomerular and podocyte damage were all alleviated. The combination therapy exhibited the most optimal outcome than the single medication. Based on these results, the combination treatment is the only therapeutic method in subsequent experiment.

### The concentration selection of adriamycin, icariin, and DEX in vitro

**Fig. 2:**
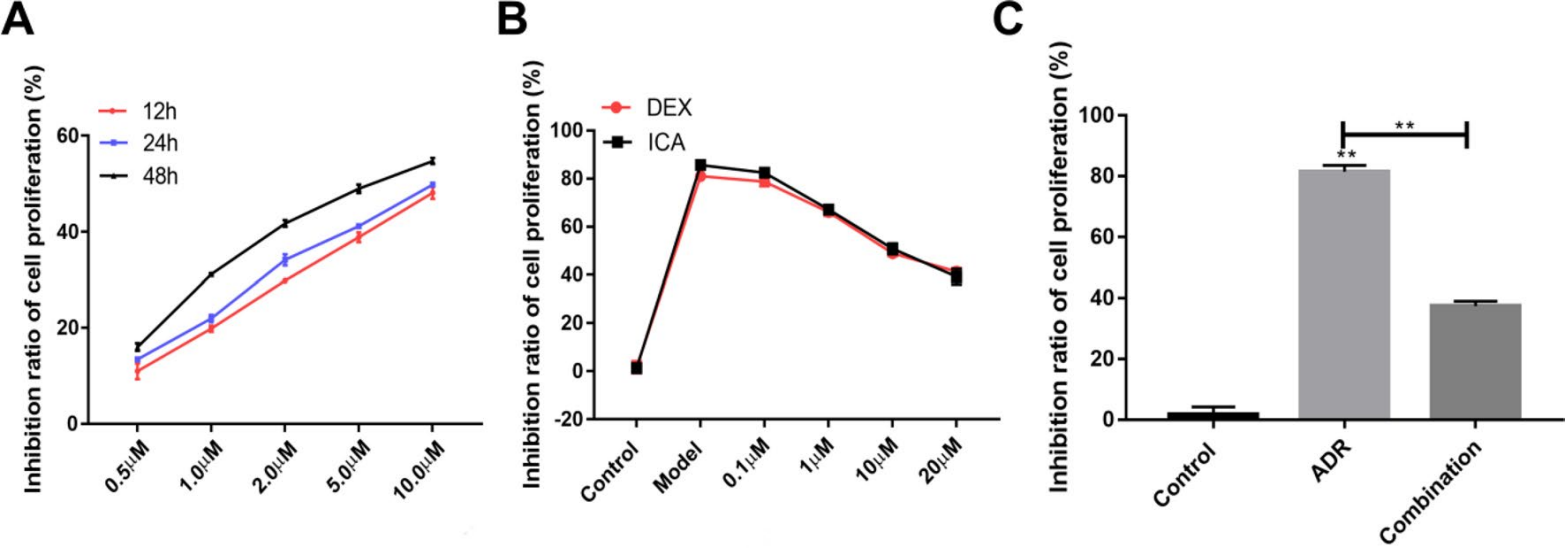
Establishment of podocytes injury and drug concentration election in vitro*.* **A** Cytotoxicity of ADR to podocytes; **B** the effect of icariin and DEX on ADR-induced podocytes injury model; **C** the therapeutic effect of icariin and DEX combination therapy on podocytes injury model

The podocytes apoptosis was gradually increased with time and concentration of ADR treatment. According to the LC50 results, the selected ADR concentration in subsequent experiments for an incubation time of 24 h was 10 μmol/L (Fig. 2A). On the other hand, DEX and ICA increased podocyte cell viability. According to the results of MTT, the selected concentration of DEX and ICA was 10 μM, for an incubation time of 24 h (Fig. 2B). It was found that ADR-induced podocyte apoptosis was significantly reduced in ICA and DEX combination treatment (Fig. 2C).

### Icariin enhanced the effect of DEX on podocytes autophagy

**Fig. 3:**
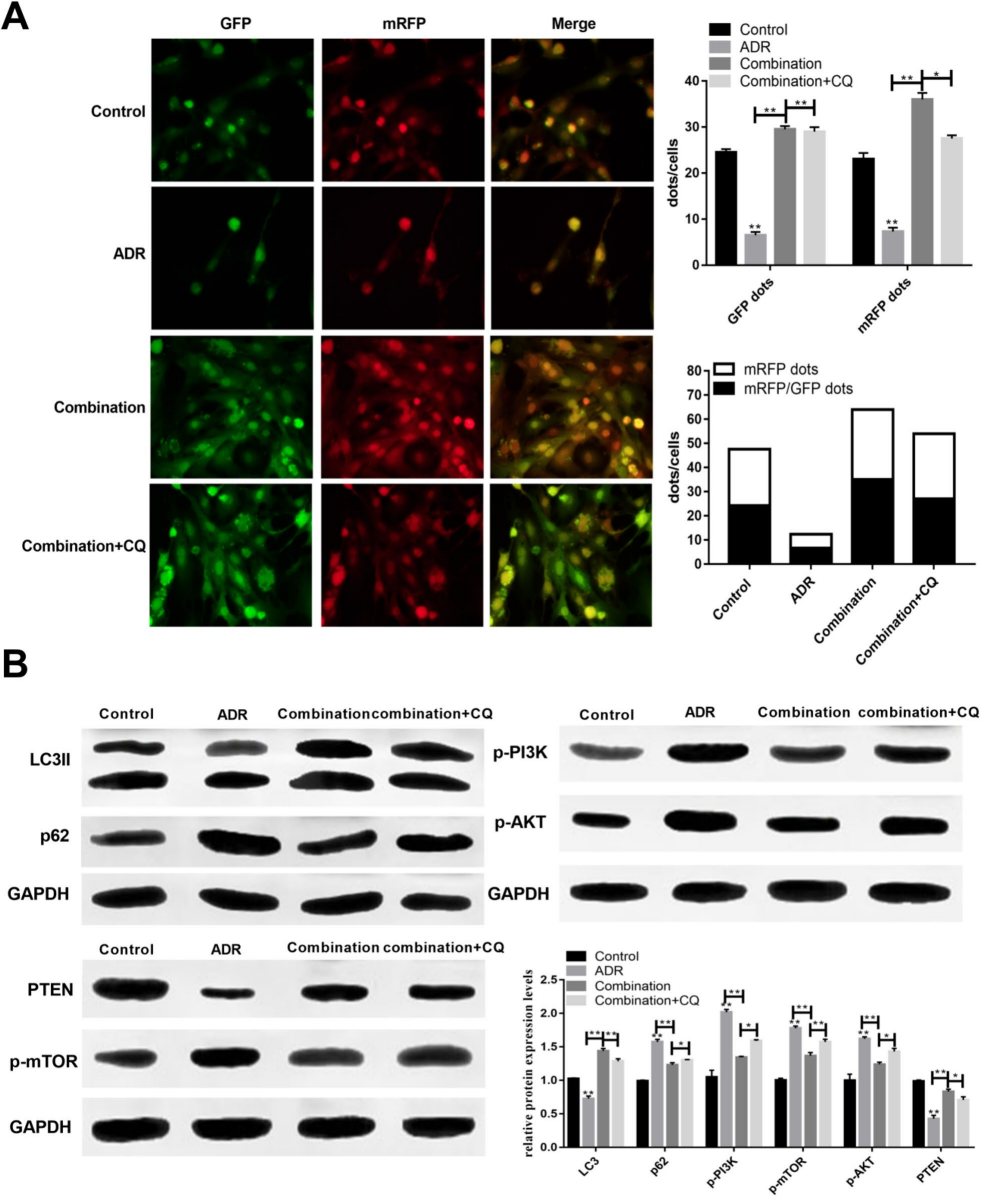
The action mechanism of combination therapy on podocytes injury. **A** Combination therapy enhanced the autophagy flux in podocytes injury; **B** combination therapy regulated the autophagy of podocytes via PI3K/AKT/mTOR signal

The apoptosis and survival of podocytes are closely related to their autophagy. To investigate the effect of combination therapy on podocyte autophagy, we used mRFP-GFP-LC3 tandem adenovirus to infect podocytes and observe autophagy. Compared with control, ADR reduced the presence of mRFP (red light) and mRFP/GFP (yellow dots) which means the inhibition of autophagy in podocytes. Compared with the model group, the combination treatment significantly increased the number of red and yellow dots. Compared with the combination treatment, the combination + CQ treatment reduced the number of red dots and increased the number of yellow dots. Together, it implied that CQ inhibited the formation of autophagy lysosomes, reduced the quenching of GFP and autophagic flux (Fig. 3A). Similarly, the changes of LC3 and p62 protein levels were consistent with the results of transfection assay. In the ADR-induced model group, the protein expression of LC3II was inhibited while p62 expression was increased. However, the combination and combination + CQ reversed the expression of LC3II and p62 (Fig. 3B). Remarkably, the downregulated PTEN protein expression and upregulated p-AKT, p-PI3K, and p-mTOR expression were observed in cells following ADR treatment. On the contrary, the combination therapy alleviated these effects (Fig. 3B). ADR activates PI3K/AKT/mTOR signal and inhibits podocyte autophagy. The combination therapy suppressed these pathways and increased podocyte autophagy.

## Discussion

Nephrotic syndrome is a common glomerular dysfunction disease in clinical nephrology. Its main pathological manifestation is the increase of glomerular basement membrane permeability. Patients can be accompanied by clinical symptoms such as abnormal lipid metabolism, edema, urinary protein, hypercoagulation, hypoproteinemia, etc. The occurrence of these clinical manifestations in patients may lead to hypercoagulable blood, which easily causes fibrin deposition in the kidney and microcirculation coagulation in the kidney, and this will cause further damage to the glomerulus [22]. The dysfunction or disorder of podocytes, glomerular basement membrane, and endothelial cells affects the glomerulus' structure and function. Among them, the apoptosis of podocytes is the main reason to the pathology of glomerular disease.

Autophagy can prevent podocyte damage from hyperglycemia, hypoxia, aging, and cancer [23]. A previous study reported that activated podocyte autophagy could alleviate ADR-induced renal injury in vivo and in vitro [24]. Restoring podocyte autophagy is a self-protection mechanism of podocytes. Studies have found that inhibiting HDAC6 can restore podocyte autophagy and reduce AGE damage to podocytes [23]. Paecilomyces cicadae-fermented Radix astragali can enhance the autophagy of podocytes induced by high glucose, enhance cell viability, and reduce apoptosis [25]. Histone deacetylase protects podocytes from damage by regulating inflammation, autophagy, and insulin resistance [26]. Hepatocyte growth factor regulates the PI3K/Akt-GSK3 sh-TFEB axis and protects diabetic nephropathy through the podocyte autophagy-lysosome pathway [27]. Overexpressed Linc 4930556M19Rik inhibits podocyte apoptosis, fibrosis, and inflammation induced by high glucose in diabetic nephropathy through miR-27a-3p/Metalloproteinase 3-axis [28]. In diabetic nephropathy, downregulation of SETD6 protects podocytes from apoptosis and mitochondrial dysfunction induced by high glucose and palmitic acid by activating the Nrf2-Keap1 signaling pathway [29].

The same results were also observed in our study. The decreased contents of proteinuria, Scr and BUN, reduced glomerular damage, and increased expression of podocin were found in rat model following ICA + DEX treatment. Also, ICA enhanced podocyte autophagy ability activated by DEX and reduced cell apoptosis in vitro. On the contrary, CQ reduces the activation of podocyte autophagy induced by DEX and ICA. Our study confirmed that ICA could enhance DEX-induced podocyte autophagy and reduce the dose of hormone drugs. Similarly, in studies in which rat cardiomyocytes fight doxorubicin-induced cardiotoxicity, ICA has been shown to promote the lipidation rate of autophagy markers beclin-1 and LC3 and restore the physiological activation levels of protective autophagy [30]. This suggests that our study is consistent with the study of Scicchitano et al. [30], which lays the foundation for the mechanism by which ICA treats nephrotic syndrome and other diseases.

## Conclusion

ICA can relieve the signs of kidney Yang deficiency caused by DEX treatment and enhance the therapeutic effect of DEX in nephrotic syndrome. ICA and DEX’s combination therapy activates podocyte autophagy by inhibiting PI3K/AKT/mTOR signal pathway.

## Data Availability

The data are available from the corresponding author on reasonable request.
